# Incidence of azole resistance among clinical isolates of *Candida parapsilosis*: Results from the French nationwide multicenter prospective study “ReCap” 2022-2024

**DOI:** 10.1016/j.nmni.2026.101738

**Published:** 2026-03-13

**Authors:** Arnaud Fekkar, Marion Blaize, Sophie Cassaing, Boualem Sendid, Grégoire Pasquier, Sébastien Imbert, Christophe Hennequin, Françoise Botterel, Florent Morio, Jean Menotti, Solène Le Gal, Eloise Bailly, Marie-Elisabeth Bougnoux, Antoine Huguenin, Christine Bonnal, Milène Sasso, Aliosha Feuss, Arnaud Jabet, Alexandre Godmer, Renaud Piarroux, Anne-Cécile Normand

**Affiliations:** a AP-HP, Groupe Hospitalier La Pitié-Salpêtrière, Parasitologie Mycologie, F-75013, Paris, France; b National Reference Centre « INuSuAle » (Digital Identification, Surveillance, Alert), La Pitié-Salpêtrière site, associated to the NRC for invasive Mycoses and Antifungal, France; c Centre Hospitalier Universitaire de Toulouse, Faculté de Santé, Hôpital Purpan, Parasitologie-Mycologie, Toulouse, France; d CHU Lille, Institut de Microbiologie, Service de Parasitologie-Mycologie, INSERM U1285, CNRS UMR 8576, Université de Lille, Lille, F-59000, France; e Laboratoire de Parasitologie-Mycologie, CHU de Montpellier, Université de Montpellier, CNRS, IRD, MiVEGEC, Montpellier, France; f Centre Hospitalier Universitaire de Bordeaux, Service de Parasitologie - Mycologie, F-33075, Bordeaux, France; g AP-HP, Groupe Hospitalier Saint-Antoine, Parasitologie Mycologie, F-75013, Paris, France; h AP-HP, Groupe Hospitalier Henri Mondor, Parasitologie Mycologie, F-94000, Créteil, France; i Nantes Université, CHU Nantes, Cibles et Médicaments des Infections et de l'Immunité, IICiMed, UR 1155, F-44000, Nantes, France; j Hospices Civils de Lyon et Université Claude Bernard-Lyon 1, Hôpital de la Croix-Rousse, Service de Parasitologie et de Mycologie Médicale, F-69004, Lyon, France; k CHU de Brest, Laboratoire de Parasitologie-Mycologie, F-29609, Brest, France; l Univ Brest, Univ Angers, Infections Respiratoires Fongiques, F-29238, Brest, France; m CHU de Dijon, Laboratoire de Parasitologie Mycologie, F-21000, Dijon, France; n AP-HP, Groupe Hospitalier Necker-Enfants Malades, Parasitologie Mycologie, F-75013, Paris, France; o CHU de Reims, Laboratoire de Parasitologie Mycologie, F-51100, Reims, France; p APHP, Hôpital Bichat Claude Bernard, Parasitologie Mycologie, F-75018, Paris, France; q Laboratoire de Parasitologie-Mycologie, CHU Nîmes, Université de Montpellier, CNRS, IRD, MiVEGEC, Montpellier, France; r Sorbonne Université, INSERM, U1135, Centre d’Immunologie et des Maladies Infectieuses, Cimi-Paris, Paris, France; s AP-HP.Sorbonne Université, Hôpital Saint-Antoine, Département de Bactériologie, Paris, France

**Keywords:** Emergence, Fluconazole, Voriconazole, Posaconazole, Cluster, Fungal infection, Multidrug resistance, Global health

## Abstract

**Background:**

Fluconazole-resistant isolates of *Candida parapsilosis* have emerged worldwide in recent years.

**Objective:**

Our objectives were to get an overview of the French epidemiology, to determine the incidence of azole resistance and to assess the genetic relationship between isolates.

**Methods:**

We initiated a prospective national multicenter study (ReCap - Resistance of *Candida parapsilosis* to azole drugs). All *C. parapsilosis* isolates routinely identified in 15 French hospital mycology laboratories were included over a period of 6 to 20 months (depending on the center), regardless of the type of specimen and hospitalization unit. Isolates were tested for fluconazole susceptibility using a gradient diffusion method. Non-susceptible isolates (MIC ≥4 mg/L) and a random selection of susceptible isolates were subjected to further analysis (MICs determination by EUCAST method and microsatellite genotyping).

**Results:**

Between May 2022 and February 2024, a total of 2,602 isolates were collected from 1,830 patients. Among these, 392 isolates (15.1%) from 184 patients (10%) were found to be resistant (MIC >4 mg/L). Percentage of patients colonized or infected by a resistant isolate ranged from 0 to 54.4% across centers. Genotyping analysis performed on 1,320 isolates showed high diversity among susceptible isolates and indicated that two fluconazole-resistant clusters are circulating in different hospitals of the Paris area. Cross-resistance occurred frequently with voriconazole resistance observed in 62.9% (73/116) of fluconazole-resistant isolates.

**Conclusion:**

In France, the frequency of resistance to fluconazole reaches 10% of patients harboring *C. parapsilosis*, with very significant differences between regions. The Paris area is affected by two epidemic clusters involving many patients.

## 1 Introduction

*Candida parapsilosis* is a common commensal cutaneous yeast as well as an opportunistic pathogen responsible for a range of infections, particularly in patients with underlying conditions and/or risk factors such as ICU hospitalization or immunocompromised situation [1]. Invasive infections range from bloodstream infections (i.e. candidemia) to deep-seated infections in organs and tissues. The first descriptions of outbreaks of *C. parapsilosis* infection involved environmental reservoirs or hand contamination of caregivers and were published in the late 1970s [2].

Antifungal agents of the azole class, especially fluconazole and voriconazole have been the mainstay in the treatment of *C. parapsilosis* infections due to their efficacy, availability, interesting cost-effectiveness ratio, and relatively low toxicity as well as this species’ lesser susceptibility toward echinocandins. In the 2000s, the first cluster cases of fluconazole-resistant *C. parapsilosis* infections were observed [3]. For several years now, a global phenomenon has emerged with the widespread of fluconazole-resistant *C. parapsilosis* isolates, often responsible for epidemics in hospitals, particularly in intensive care units [[4], [5], [6], [7]]. The prevalence of resistance is taking on very significant and worrying proportions in some countries, reaching or even exceeding 50% [8,9].

Molecular mechanisms underpinning azole resistance in *C*. *parapsilosis* involve alterations in the targeted enzyme, lanosterol 14-alpha-demethylase, encoded by the *ERG11* gene or other mechanisms [10,11]. Among the alterations of Erg11, the most widely reported is Y132F, which alters the structure of the enzyme, reducing the binding affinity of azoles and making them less effective. In our institution located in Paris, France we have also been facing an epidemic due to resistant isolates of *C. parapsilosis* harboring the Y132F alteration [12]. Another hospital of the Paris area was concerned by such an epidemic [13].

In this context, we decided to initiate the ReCap (Resistance of *Candida parapsilosis* to azoles drugs) study - a prospective, national, multicenter study designed to describe the epidemiology of *C. parapsilosis* resistance to azole drugs in hospitals. We aimed to determine the percentage of resistance among all *C. parapsilosis* isolates get in hospital mycology laboratories, to look for the existence of one or more clones, and to investigate the genetic relationships between clinical isolates.

## 2 Material and methods

***Participating centers and isolates collection.*** Fifteen medical mycology laboratories settled in teaching hospitals were asked to participate in the study. Each center ensured the inclusion of all *C. parapsilosis* isolates routinely identified from clinical specimens received to the laboratory for a period of at least 6 months. All specimen types and all clinical departments were involved. For the same patient, isolates obtained either on different days, or on the same day but from different anatomical sites, were all included.

***Screening of isolates for fluconazole resistance and determination of susceptibility profile.*** The Minimal Inhibitory Concentrations (MIC) of fluconazole was determined for all isolates using a concentration gradient strip method (Etest®, Biomérieux®). For convenience, the intermediate values of the MICs obtained in the Etest® were rounded up to the nearest value existing in the reference technique procedure. We decided to apply the values proposed by the EUCAST for the interpretation of the MICs value. So, the thresholds were as follows: susceptible (MIC ≤2 mg/L), intermediate (MIC = 4 mg/L), or resistant (MIC >4 mg/L). Non-susceptible (i.e. resistant and intermediate) isolates and a random panel of susceptible isolates were centralized in our institution, the La Pitié-Salpêtrière Hospital (referred to throughout the manuscript as the “coordinating laboratory”), for further analysis. These latter include the determination of the MICs of fluconazole, voriconazole, posaconazole and isavuconazole using the EUCAST reference method and molecular typing.

***Molecular analysis.*** Microsatellite genotyping was performed following the protocol published by Diab-Elschahawi et al. in 2012 [14] using six microsatellites regions. Graphical representation of the microsatellites clustering was obtained using the phyloviz freeware (https://www.phyloviz.net/).

***Genetic diversity analysis.*** The distribution of genotypic profiles between resistant and susceptible isolates was compared using a Pearson's chi-square test of independence. Because several profiles had low frequencies (<5 isolates), the p-value was estimated by Monte Carlo simulation (2,000 random replicates) to ensure validity of the test. The null hypothesis assumed identical profile distributions between groups. To analyze cross-resistance, odds ratios (OR) with 95% confidence intervals (CI) were calculated using Fisher's exact test. For voriconazole, the exact mid-P test was used due to sparse data (cell count <5). All analyses were performed using R 4.3.2 (exact2x2 package).

## 3 Results

**Fig. 1:**
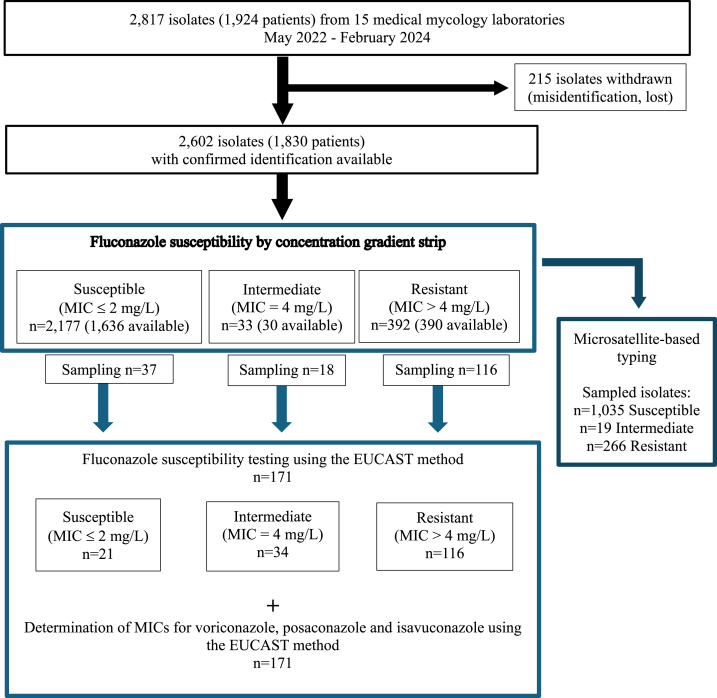
Flowchart of the “ReCap” study, a French nationwide multicenter prospective study for determining the incidence of azole resistance in *Candida parapsilosis*.

***Patients and isolates.*** A total of 15 medical mycology laboratories settled in teaching hospital in various cities (Brest, Lyon, Paris (n = 4), Lille, Nîmes, Toulouse, Dijon, Reims, Bordeaux, Nantes, Montpellier, and Créteil) in mainland France took part in the study. Between May 2022 and February 2024, participating centers ensured the inclusion of 2,817 *C. parapsilosis* isolates routinely identified from clinical specimens from 1,924 different patients (Fig. 1 and Supplemental Data Fig. S1). Among these isolates, 215 were excluded from the study because they were either lost during subculture, contaminated by other species, or finally identified as non-*C. parapsilosis* isolates. Period of inclusion varies from 6 to 20 months depending on each center's capabilities. Determination of fluconazole MIC by the Etest® was performed for 1,442 isolates at the corresponding center, in which case only the non-susceptible isolates and a random selection of susceptible isolates were sent to the coordinating laboratory. A total of 1,160 isolates were not tested locally and were therefore sent to the coordinating laboratory for MIC determination by the Etest®.

**Fig. 2:**
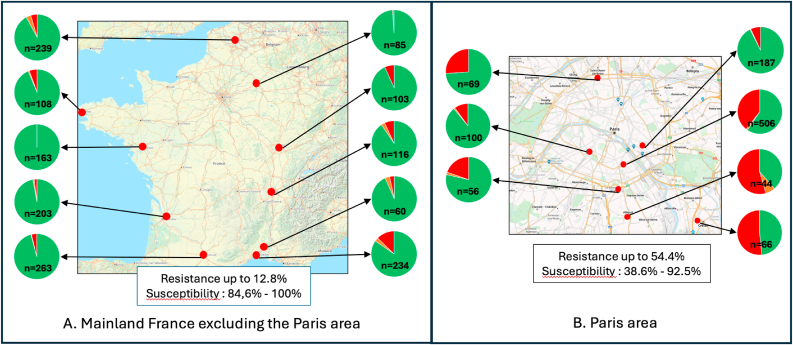
Distribution of *Candida parapsilosis* isolates collected from 15 medical mycology laboratories (17 hospital) in France between May 2022 and February 2024, according to their susceptibility to fluconazole as determined by Etest®. Green: susceptible isolates (MIC ≤2 mg/L); orange: intermediate susceptibility (MIC = 4 mg/L); red: resistant isolates (MIC >4 mg/L). A: mainland France excluding the Paris region. B: Paris region.

***Incidence and geographical distribution of fluconazole resistance.*** A total of 2,602 isolates (sampled from 1,830 different patients) were tested, of which 392 (184 patients; 10%) were found “resistant” to fluconazole and 33 (32 patients) “intermediate” using the Etest® method. Thus, the overall incidence of non-susceptibility of *C. parapsilosis* per patient was 11.8% (216/1,830) but with significant geographical disparities (Fig. 2). All isolates sent to our institution were checked for their identification by MALDI-ToF mass spectrometry (Bruker Microflex and MSI-2 database) before their MICs were determined using the Etest® diffusion gradient method. We then constituted a panel of 171 isolates (37 susceptible, 18 intermediate and 116 resistant to fluconazole) and determined the fluconazole MIC by the EUCAST method.

**Fig. 3:**
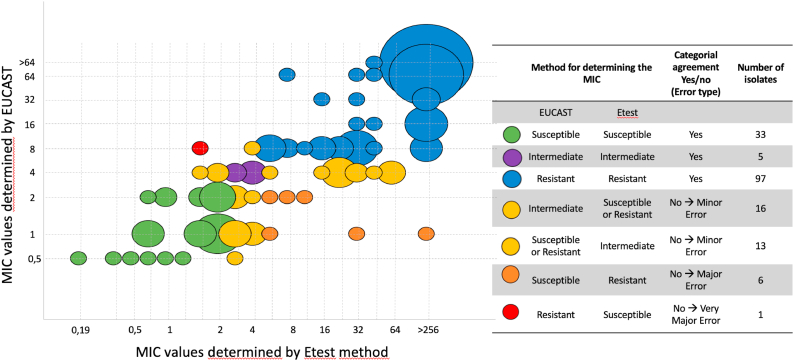
Comparison of fluconazole minimum inhibitory concentrations (MICs) determined by the Etest® method and the EUCAST (European Committee on Antimicrobial Susceptibility Testing) method for 171 *Candida parapsilosis* isolates.

Not all resistant isolates were tested using the EUCAST method, particularly because a large proportion of them were identified as belonging to different clonal populations (cf *infra*). The overall categorical agreement between the two methods was 78.9%, with 17.0% minor errors (“susceptible” instead “intermediate” or “resistant” instead “intermediate”; n = 29), 3.5% major errors (i.e. resistant by Etest® and susceptible by EUCAST; n = 6), and 0.6% very major errors (i.e. susceptible by Etest® and resistant by EUCAST; n = 1) (Fig. 3). Of note, the seven isolates exhibiting major errors and very major errors were re-tested using both techniques, producing similar results. If isolates with intermediate results according to Etest® and/or EUCAST were excluded from the analysis, the categorical agreement between Etest® and EUCAST increased to 94.9% (130/137).

***Susceptibility to other azole drugs*.** The 171 isolates whose MICs to fluconazole had been determined both by Etest® and by the EUCAST method were also tested for their susceptibility to voriconazole, posaconazole and isavuconazole (Table 1). Of note was a link between high fluconazole MIC values and resistance to voriconazole as 91.9% (68/74) of the voriconazole-resistant isolates (i.e. MIC >0.25 mg/L) had a fluconazole MIC ≥32 mg/L. Furthermore, a strong association between fluconazole and voriconazole resistance was observed and confirmed by statistical analysis, with voriconazole resistance observed in only 1.8% (1/55) of fluconazole-susceptible/intermediate isolates compared to 62.9% (73/116) of fluconazole-resistant isolates (OR = 89.72, 95% CI: 14.34-3657.08, P < 0.001). No significant association was found with posaconazole resistance (MIC >0.06 mg/L) (OR = 1.52, 95% CI: 0.67-3.68, P = 0.347). Resistance rates were 20.0% (11/55) and 27.6% (32/116) in the fluconazole-susceptible/intermediate and fluconazole-resistant groups, respectively.

**Table 1:** Distribution of Minimal Inhibitory Concentrations determined by EUCAST method for voriconazole, posaconazole and isavuconazole for 171 *Candida parapsilosis* isolates according to their susceptibility profiles to fluconazole. According to EUCAST, voriconazole resistance is defined as an MIC greater than 0.25 mg/L, while posaconazole resistance is defined as an MIC greater than 0.06 mg/L.

Susceptibility to fluconazole	Azole drug	Minimal Inhibitory Concentration (mg/L)									
		0.016	0.03	0.06	0.125	0.25	0.5	1	2	4	8
**Susceptible (MIC ≤ 2 mg/L) n = 21**	voriconazole	2	5	10	4	0	0	0	0	0	0
	posaconazole	8	3	2	3	5	0	0	0	0	0
	isavuconazole	2	14	5	0	0	0	0	0	0	0
**Intermediate (MIC = 4 mg/L) n = 34**	voriconazole	0	5	16	9	3	1	0	0	0	0
	posaconazole	27	4	0	3	0	0	0	0	0	0
	isavuconazole	0	27	4	3	0	0	0	0	0	0
**Resistant (MIC>4 mg/L) n = 116**	voriconazole	0	6	6	22	9	9	10	34	17	3
	posaconazole	36	32	16	13	17	2	0	0	0	0
	isavuconazole	2	20	26	41	20	0	4	2	1	0

**Fig. 4:**
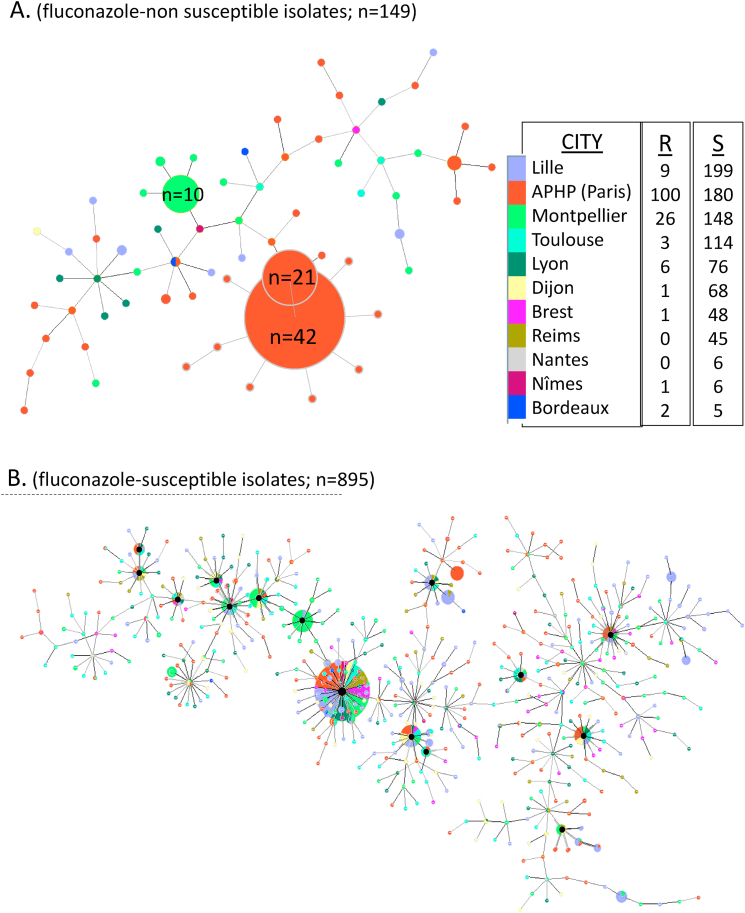
Minimum spanning tree showing the analysis of the genotypic diversity determined by microsatellite method for 1,044 isolates of *Candida parapsilosis* collected in 15 French mycology laboratories according to their susceptibility to fluconazole. **A.** Distribution of the fluconazole non-susceptible isolates (resistant or intermediate; n = 149). Two resistant clones circulating in several hospitals in the Paris area were observed in 34.2% (63/184) of patients harboring non-susceptible isolates. Another resistant clone was found in the city of Montpellier in 42.5% (10/22) of the patient. **B.** Distribution of the fluconazole susceptible isolates (n = 895). Genetic diversity was higher among susceptible isolates, although several profiles circulating in different hospitals were observed and especially one circulating between 11 hospitals and representing 4.9% (44/895) of the patients carrying a susceptible isolate.

***Genotyping analysis*.** A total of 1,320 isolates were submitted to microsatellites analysis: 266 were fluconazole-resistant, 19 were intermediate, and 1,035 were fluconazole-susceptible. Fig. 4 shows the results for a total of 1,044 unique profile/patient combinations, i.e. not considering replicates per patients. The distribution of genotypic profiles differed significantly between resistant and susceptible isolates (p < 0.0005 by Chi-square test).

Among resistant isolates, two clones circulating in the Paris area accounted for 59.8% of isolates (159/266), being present in 34.2% of patients (63/184). In addition, 11 other genotype profiles were closely related to these two clones. Another clone was found in Montpellier, where 45.5% (10 out of 22) of patients harbored resistant isolates with the same genetic profile.

Genetic diversity was higher among susceptible isolates, as shown by a greater number of profiles and higher Shannon (H′ 5.82523 vs 2.58831) and Simpson (1−D 0.00640668 vs 0.199107) index (data not shown). However, we observed several identical profiles which circulated in different hospitals, and especially one circulating clone common to 11 hospital centers and representing 4.9% of patients (44/895) harboring a fluconazole-susceptible isolate.

## 4 Discussion

The global emergence of azole-resistant *C. parapsilosis* is an important issue from a human health perspective and one that has worldwide implications [8]. In this article, we report on the epidemiology of *C. parapsilosis* in France on a national scale and provide original data of the incidence of resistance to azole drugs in hospitals. We uncover the existence of fluconazole-resistant clonal populations in several hospitals in a common geographical area centered around the city of Paris. Our approach, which is based on the inclusion of both colonizing and infecting isolates, enables us to obtain a global and more realistic incidence. Limitations of this study include the analysis of isolates genetic diversity based on a simple microsatellite approach. The study's significance is also limited by the lack of demographic and clinical data, such as the nature of the sample, the patient hospitalization unit or the therapies used. Another limitation is that the inclusion period varied from center to center. However, the important point is that, regardless of the period, the search for resistance encompasses all isolates, making it possible to measure the actual incidence. These approaches were thus adequate to meet the study objectives and capture the major resistance patterns observed.

While the origin of the worldwide emergence of *C. parapsilosis* resistance to antifungals remains a mystery, several factors may have contributed to the development of azole resistance in this common yeast. One major factor might be the use, overuse or misuse of azole antifungals, both in clinical and environmental/agricultural settings. Prolonged and repeated exposure to these drugs exerts selective pressure on the fungal population, favoring the survival and proliferation of resistant strains. Additionally, the widespread use of azole antifungals in agriculture has been implicated in the environmental reservoir of azole-resistant *Aspergillus fumigatus* and other *Candida* spp isolates, potentially contributing to the dissemination of resistance in healthcare settings [15,16]. Finally, it would be of great interest to determine whether the resistance mechanism offers a selective advantage to the mutated isolate compared to the wild-type genotype, which could explain its persistence in the hospital environment and clonal spread [17].

We describe the fact that a large number of patients harbor resistant isolates belonging to two closely related clones. More comprehensive genomic analyses would improve our understanding of how the clones spread in the Paris area. Moreover, analyzing isolates detected in non-hospital laboratories would provide insight into how widespread resistance is in the general population. Interestingly, Brassington et al. demonstrated a high degree of genetic similarity between, on one hand, *C. parapsilosis* isolates responsible for outbreaks in Germany and Canada, and on the other hand, those found in countries as diverse as Kuwait, South Korea, and Turkey [18]. Therefore, a study incorporating isolates from other countries would be highly relevant.

To further this work, sequencing of the *ERG11* gene would be useful, not only for resistant isolates but also to investigate the Etest®/EUCAST discrepancies. Most studies reporting outbreaks of fluconazole-resistant *C. parapsilosis* isolates have pointed to the presence of the Y132F mutation [4,6,13,19]. This alteration is interesting because it might even be considered a surrogate marker for clonality and the transition of resistant isolates to endemicity [6]. As a preliminary step, we have started such an analysis. In a sample of 98 genetically distinct (i.e., non-clonal) resistant isolates, the Y132F alteration was found in 52 isolates (53%) (including those representing the clones) while the wild-type *ERG11* sequence was found in 44 isolates (44.9%) (data not shown). Moreover, the Y132F alteration is not in itself sufficient to explain the high level of resistance among the clonal population in the Paris area (MIC>256 mg/L) as this alteration alone causes only a modest increase in the MIC for fluconazole and voriconazole [20]. The upregulation of efflux pumps, which actively expel azoles from the fungal cell, represents another common mechanism of resistance [21]. Others complex mechanisms known in others *Candida species* and recently described in *C. parapsilosis* might also be involved, such as copies number variations (CNV) [10,11]. Thus, the existence of one or more of these different resistance mechanisms could explain the variable level of resistance between isolates, the high resistance phenotype of clonal isolates and should be investigated as part of the continuation of this study. Finally, the comprehensive and detailed knowledge of the various molecular mechanisms involved in resistance phenomena may explain cross-resistance between azole drugs and could even be predictive of virulence [22].

The manufacturer of the Etest® recommends interpretation based on CLSI, but we primarily used the EUCAST cut-offs, which are not very different anyway. Moreover, it is well established that the MIC values determined by the EUCAST reference method and the commercial Etest® gradient concentration strip method are very well correlated [23]. Nevertheless, complete categorical agreement is not achieved and the use of one or the other of the methods will consequently give significantly different results. To us, this difference seems marginal. In the context of a large surveillance study, it is important to have a simple, practical alternative to EUCAST, available to as many laboratories as possible for routine testing. Another pitfall is that for fluconazole there is a concentration value of 4 mg/L for which the yeast is considered neither susceptible nor resistant. Thus, most discrepancies were observed among isolates whose fluconazole MICs were close to the threshold and were reinforced by the existence of this “intermediate” category. The same applies to the CLSI values recommended for interpreting the results obtained by Etest®: these are values within a range of concentrations strictly greater than 2 mg/L and strictly less than 8 mg/L, which is more or less the same as the EUCAST criteria.

As the incidence of azole-resistant strains is on the rise, understanding the underlying mechanisms, implementing effective surveillance, and developing strategies to optimize antifungal use are imperative for mitigating the impact of this emerging public health challenge. As is often the case in medical mycology, it is essential to know and monitor the local epidemiology, i.e. the distribution of species, the level of resistance to antifungal drugs and their evolution over time in the environment in which we work. The results of this study highlight this need: some areas are already facing a high incidence, leading to a change in therapeutic strategy in some departments, while some unaffected areas should put this species under surveillance. Finally, addressing the environmental aspect of resistance requires collaboration between the health and agricultural sectors to think transversally about the use of azole antifungals, particularly at a time when this problem may emerge in other common species such as *Candida tropicalis* [24].

In conclusion, *Candida parapsilosis* resistance to azole antifungals is a pressing medical problem that demands attention and concerted efforts from the medical and research communities. Our work indicates that in France, the frequency of resistance to fluconazole reaches 10.1% of patients harboring *C. parapsilosis*, with very significant differences between regions. The Paris area is affected by two epidemic clusters involving many patients.

## CRediT authorship contribution statement

**Arnaud Fekkar:** Writing – review & editing, Writing – original draft, Visualization, Validation, Supervision, Resources, Project administration, Methodology, Investigation, Funding acquisition, Formal analysis, Data curation, Conceptualization. **Marion Blaize:** Writing – review & editing, Writing – original draft, Methodology, Investigation, Data curation, Conceptualization. **Sophie Cassaing:** Resources, Investigation. **Boualem Sendid:** Resources, Investigation. **Grégoire Pasquier:** Resources, Investigation. **Sébastien Imbert:** Resources, Investigation. **Christophe Hennequin:** Resources, Investigation. **Françoise Botterel:** Resources, Investigation. **Florent Morio:** Resources, Investigation. **Jean Menotti:** Resources, Investigation. **Solène Le Gal:** Resources, Investigation. **Eloise Bailly:** Resources, Investigation. **Marie-Elisabeth Bougnoux:** Resources, Investigation. **Antoine Huguenin:** Resources, Investigation. **Christine Bonnal:** Resources, Investigation. **Milène Sasso:** Resources, Investigation. **Aliosha Feuss:** Writing – review & editing, Conceptualization. **Arnaud Jabet:** Writing – review & editing, Resources, Investigation, Formal analysis. **Alexandre Godmer:** Writing – review & editing, Formal analysis. **Renaud Piarroux:** Writing – review & editing, Investigation, Formal analysis, Conceptualization. **Anne-Cécile Normand:** Writing – review & editing, Writing – original draft, Visualization, Validation, Methodology, Investigation, Formal analysis, Data curation, Conceptualization.

## Declaration of competing interest

The study did not benefit from any specific financial support and was supported with internal funding. Part of the study results was presented as an oral communication during the ECCMID Congress (2024, April 29, Barcelona, Spain).
