# Supplementary material for: Incidence of azole resistance among clinical isolates of Candida parapsilosis: Results from the French nationwide multicenter prospective study “ReCap” 2022-2024
Source: New Microbes New Infect. 2026 Mar 13;71:101738. doi: 10.1016/j.nmni.2026.101738 (PMC13014649; doi:10.1016/j.nmni.2026.101738)
Supplement: Multimedia component 1 [file mmc1.pptx]

## Slide 1
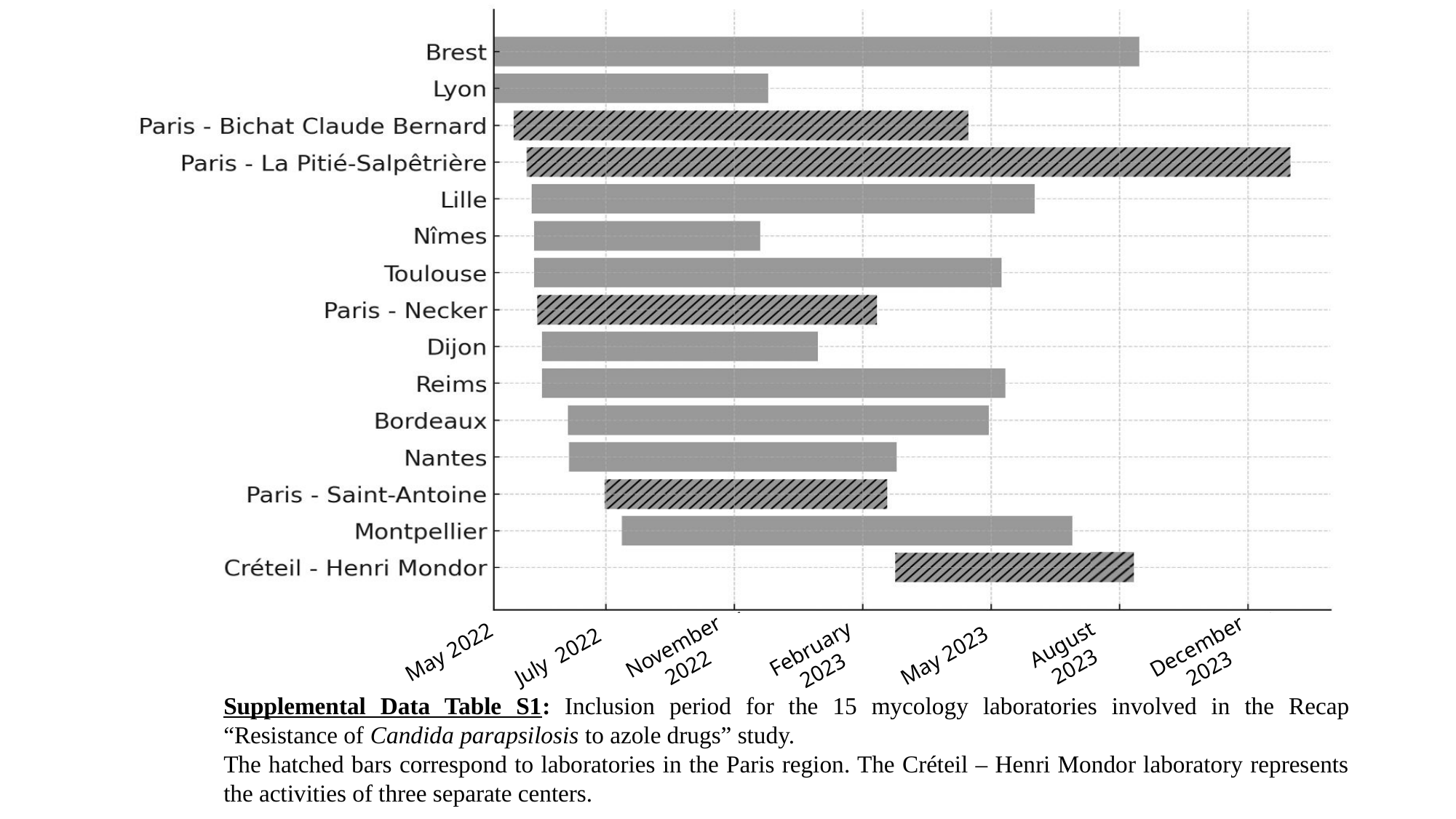

August 2023
November 2022
December 2023
February 2023
May 2022
May 2023
July 2022
Supplemental Data Table S1: Inclusion period for the 15 mycology laboratories involved in the Recap “Resistance of Candida parapsilosis to azole drugs” study.
The hatched bars correspond to laboratories in the Paris region. The Créteil – Henri Mondor laboratory represents the activities of three separate centers.
